# P2X and P2Y Receptors—Role in the Pathophysiology of the Nervous System

**DOI:** 10.3390/ijms151223672

**Published:** 2014-12-18

**Authors:** Kamila Puchałowicz, Maciej Tarnowski, Irena Baranowska-Bosiacka, Dariusz Chlubek, Violetta Dziedziejko

**Affiliations:** 1 Department of Biochemistry and Medical Chemistry, Pomeranian Medical University, 72 Powstańców Wielkopolskich Street, Szczecin 70-111, Poland; E-Mails: kamila.puchalowicz@op.pl (K.P.); dchlubek@pum.edu.pl (D.C.); viola@pum.edu.pl (V.D.); 2 Department of Physiology, Pomeranian Medical University, 72 Powstańców Wielkopolskich Street, Szczecin 70-111, Poland; E-Mail: maciejt@pum.edu.pl

**Keywords:** Alzheimer’s disease, ATP, depression, epilepsy, glioma, multiple sclerosis, neurodegenerative disease, neuropathic pain, P2X receptors, P2Y receptors

## Abstract

Purinergic signalling plays a crucial role in proper functioning of the nervous system. Mechanisms depending on extracellular nucleotides and their P2 receptors also underlie a number of nervous system dysfunctions. This review aims to present the role of purinergic signalling, with particular focus devoted to role of P2 family receptors, in epilepsy, depression, neuropathic pain, nervous system neoplasms, such as glioma and neuroblastoma, neurodegenerative diseases like Parkinson’s disease, Alzheimer’s disease and multiple sclerosis. The above-mentioned conditions are associated with changes in expression of extracellular ectonucleotidases, P2X and P2Y receptors in neurons and glial cells, as well as releasing considerable amounts of nucleotides from activated or damaged nervous tissue cells into the extracellular space, which contributes to disturbance in purinergic signalling. The numerous studies indicate a potential possibility of using synthetic agonists/antagonists of P2 receptors in treatment of selected nervous system diseases. This is of particular significance, since numerous available agents reveal a low effectiveness and often produce side effects.

## 1. Introduction

The role of purinergic signalling in physiology and pathology of the nervous system is currently an incredibly quickly developing field of study. Extracellular ATP is a signalling molecule of great importance in proper functioning of the nervous system. Under physiological conditions, ATP is released in small amounts by astrocytes and neurons creating synaptic connections [1]. Its concentration in the extracellular space reaches the nanomolar range [2,3]. Activity of extracellular nucleotides is mediated by P2 receptors, the family of membrane receptors for extracellular nucleotides, *i.e.*, ATP, ADP, UTP and UDP, and further divided into two subtypes—Ionotropic (P2X) and metabotropic (P2Y) receptors.

P2X receptors are fast acting (about 10 ms) ion channels permeable for cations (Na^+^, K^+^ and Ca^2+^), and ATP is their key agonist. They function in the form of homotrimers (P2X_1–7_) and heterotrimers or multimers, which differ significantly between each other in their distribution in tissues, permeability for ions, sensitivity to agonists and antagonists, as well as desensitisation [4]. Expression of all subunits of P2X receptors was observed in nervous tissue cells, and their distribution in particular regions of the nervous system is heterogeneous [5]. The best characterized mechanism underlying the activity of these receptors result from their high permeability for Ca^2+^. Activation of P2X receptors induces an increase in intracellular Ca^2+^ and depolarization wave, which leads to signal transmission. Functional interactions with other ion channels, K^+^ outflow and Na^+^ influx are also involved in P2X receptors signaling [6]. The P2X_7_ receptor is particularly interesting as its expression was reported mainly in cells within the hematopoietic lineage and in glial cells. It is also known for a relatively low affinity to ATP, and its maximal activity is achieved with concentrations occurring in pathological conditions (100–1000 µM) [5]. Its prolonged activation leads to transitive forming of pores in cellular membranes that are permeable for soluble substances up to 900 Da, which may be cytotoxic. It also induces numerous intracellular signal transduction pathways, for example the activation of PLA_2_ (phospholipase A_2_), PLD (phospholipase D), MAPK (mitogen-activated protein kinase), calcineurin or NF-κβ (nuclear factor κβ) [7].

P2Y receptors differ significantly from P2X receptors within the scope of their structure, sensitivity to agonists, as well as the activity mechanism itself. P2Y receptors belong to a family of G protein-coupled receptors and they activate a significant number of intracellular pathways and second messengers, and hence they act slower than P2X receptors that are ion channels [8]. Their structure includes seven transmembrane domains, next to three extracellular and three intracellular loops. P2Y receptors are sensitive both to purine and pyrimidine nucleotides. P2Y_1_ (ADP and ATP), P2Y_11_ (ATP), P2Y_12_ (ADP), P2Y_13_ (ADP and ATP) are selective receptors for adenine nucleotides, whereas P2Y_4_ (UTP), P2Y_6_ (UDP) and P2Y_14_ (UDP) are selective receptors for uracil nucleotides. P2Y_2_ receptor is activated both by ATP and UTP, while P2Y_14_ receptor is sensitive (apart from UDP) to UDP-glucose. Depending on the induced mechanism, these receptors can be divided into two groups: (1) “P2Y_1_-like”, which are coupled with G_q_ protein and they activate PLC (phospholipase C) (P2Y_1_, P2Y_2_, P2Y_4_, P2Y_6_, P2Y_11_); (2) “P2Y_12_-like”, which are coupled with G_i_ protein and they inhibit adenylate cyclase (P2Y_12_, P2Y_13_, P2Y_14_) [9]. Within the nervous system, they participate not only in short-term, but also in long-term activity [10].

ATP, as well as other extracellular nucleotides and their receptors, participates in such physiological processes as neurotransmission and neuromodulation [5], myelination [10,11], regulation of activity of microglia and astrocytes, as well as in inducing their responses to damaging factors [10,12]. Significant amounts of nucleotides are released into the extracellular space by activated and damaged cells in pathological condition, for example in the course of cerebral trauma and ischemia or neurodegenerative diseases. Their concentration may reach values considerably exceeding the ones observed in physiological conditions [13]. Apart from this, expression of P2 receptors and the system of extracellular ectonucleotidases may undergo certain changes. This leads to dysfunctions within the nervous tissue cells. Undoubtedly, dysfunctions of purinergic signalling participate in pathogenesis and progression of nervous system diseases. This review presents a detailed analysis concerning current data focusing on the role of receptors for nucleotides in nervous system disorders of various etiology—epilepsy, neuropathic pain, depression, neurodegenerative diseases such as Alzheimer’s disease, Parkinson’s disease and multiple sclerosis—as well as in nervous system tumours (Table 1).

## 2. Selected Conditions of the Nervous System

### 2.1. Epilepsy

Epilepsy is a chronic condition and the most common neurological disease, which is characterized by the occurrence of spontaneous seizures. It is being estimated that about 50 million people all over the world suffer from this condition—mostly small children and people after 65 years of age. The incidence of seizures are the sign of transient abnormalities in brain functioning that result from excessive and spontaneous bioelectric neural discharges. One of the reasons associated with their prevalence is the lack of balance between neurotransmitters: the excitatory one, glutamate and the inhibiting one, GABA (γ-aminobutyric acid). In many cases, reasons underlying epilepsy remain unknown. It is assumed that microglia and astrocytes, which express P1 and P2 receptors, play an important role in the development of epilepsy and they influence the level of exacerbation of seizures [14,15,16,17].

The role of purinergic receptors in pathophysiology of epilepsy (Table 1) is supported by the occurrence of seizure or its exacerbation after administering ATP analogues into the piriform cortex or ventricular system of mice [18]. During the seizure neuronal activity is increased, which leads to increased K^+^ concentration in the extracellular space. In response to changes in concentration of this cation, Panx1 channels located in hippocampal neurons and astrocytes are opened and ATP is released. Activation of ATP receptors may lead to hyperactivity of neurons and hence it may induce a positive feedback mechanism, progression of seizures and their prolonged duration. When compared with ATP, adenosine exhibits an anti-epileptic activity by means of the P1A_1_ receptor. Activation of this receptor inhibits glutamate release from presynaptic endings, as well as hyperpolarisation of the postsynaptic neurons [15]. However, activating another receptor from the P1 family—namely P1A_2A_ induces a contrary activity [14].

**Table 1:** P2 receptors in selected disorders of the nervous system.

P2R	Epilepsy	NP	Depression	AD	PD	SM	Glioma	NB
P2X_2_	[19]	-	-	-	-	-	-	-
P2X_3_/P2X_2/3_	-	[20,21,22]	-	-	-	-	-	-
P2X_4_	[23,24]	[25,26,27,28,29]	-	[30]	-	[31,32]	-	[33]
P2X_7_	[19,23,34,35,36,37]	[38,39,40]	[41,42,43,44,45,46,47,48,49,50,51]	[52,53,54,55,56,57]	[58,59,60,61]	[62,63,64,65,66,67]	[68,69,70]	[71,72,73,74,75]
P2Y_1_	-	-	-	-	-	-	[76,77]	-
P2Y_2_	-	[78]	-	[79,80,81,82,83,84]	-	-	-	-
P2Y_4_	-	-	-	[82]	-	-	-	-
P2Y_6_	-	[85,86]	-	-	-	-	-	[87]
P2Y_11_	-	[85]	-	-	-	-	-	-
P2Y_12_	-	[25,28,88]	-	-	-	[89,90]	[76]	-
P2Y_13_	-	[86]	-	-	-	-	-	-
P2Y_14_	-	[86]	-	-	-	-	-	-

AD: Alzheimer’s disease; PD: Parkinson’s disease; NB: neuroblastoma; NP: neuropathic pain; P2R: P2 receptor; SM: multiple sclerosis.

The P2X_7_ receptor is a purinergic receptor playing a crucial role in the course of epilepsy. Studies on rodents revealed an increased expression of P2X_7_ receptor in hippocampal neurons (granule cell layer of the dentate gyrus, CA1 region), the neocortex and in microglia observed after epileptic seizure (especially after status epilepticus), as well as its increased sensitivity to ATP, and as a consequence a rapid activation of microglial cells [19,23,34,35]. Studies performed on animal models also prove that the activation of this receptor may induce an increased glutamate release, intensification of epileptic seizures as well as neuronal death [19]. According to studies conducted by Kim *et al.* [36] leukocytes (mainly neutrophils) that infiltrate the tissue (frontoparietal cortex) in response to activation of microglial P2X_7_ receptors occurring during status epilepticus (pilocarpine model) are responsible for neuronal impairment. The inflow of leukocytes depends on IL-1β (proepileptic activity), which is released by microglial cells after activating P2X_7_ receptors, and which regulates the release/expression of chemokines, namely MCP-1 (monocyte chemoattractant protein 1) and MIP-2 (macrophage inflammatory protein 2), in neurons and glial cells. They induce chemotaxis of macrophages, neutrophils, T lymphocytes and dendritic cells into altered tissue, which releases proinflammatory cytokines and proteolytic enzymes, as well as generate ROS (reactive oxygen species), which leads to cellular damage [36]. Activation of P2X_7_ receptors on the surface of astrocytes and oligodendrocytes in the course of status epilepticus may lead to death of these cells [17]. Apart from IL-1β, TNF-α (tumour necrosis factor-α) is yet another cytokine of considerable significance in epilepsy, which is released by immune and nervous tissue cells within the mechanism depending on P2X_7_ receptor. In their subsequent studies, Kim *et al.* [37] stated that the agonist of P2X_7_ receptor (BzATP; 2',3'-*O*-(benzoyl-4-benzoyl)-ATP) induces overexpression of TNF-α in neurons of the region CA3 of hippocampus and as a consequence, TNF-α induces a route preventing neuronal death, and hence attenuates harmful effects of status epilepticus. Its antagonists, namely OxATP (oxidized ATP), A-438079, A-740003, exacerbate this adverse activity. Basing on studies performed by Kim *et al.* [36,37] it is possible to state that the neuroprotective mechanism depends on: (1) inhibition of neuroinflammation (frontoparietal cortex); (2) signalling pathway dependence on TNF-α and its TNFp75 receptor (tumour necrosis factor p75 receptor) (region CA3 of the hippocampus). Mechanisms of neuroprotective activity induced by antagonists and agonists, respectively of the P2X_7_ receptor have been described. All existing discrepancies in these agonists and antagonists activities most probably result from diverse location of receptors in the brain [37]. Other research revealed that the use of antagonists of the P2X_7_ receptor towards microglial cells decreases their activation, proliferation and production of IL-1β, limits the release of stimulating neurotransmitters, and prevents excitotoxicity and damage of the hippocampus [17,18,19,35,36]. Apart from the above, Engel *et al.* [19] suggest that the reduction in seizure duration is responsible for neuroprotective activity, as it considerably decreases the risk associated with the damage of neurons. When combined with traditional medications characterized by a different activity mechanism, such as lorazepam, antagonists of P2X_7_ receptor not only efficiently inhibit seizures, including epileptic status, but also shorten their duration. To sum up, mechanisms responsible for antiepileptic activity of P2X_7_ receptor’s antagonists may result from the following: (1) on the level of neurons—from inhibition of presynaptic glutamate release or excitatory postsynaptic potential; and (2) on the microglial level—from suppression of neuroinflammation induced by activated microglial cells. Obtaining a proper therapeutic result depends to a large extent on the site of administration, as well as on the time of administration [19].

Apart from increased expression of P2X_7_ receptor, what could be observed within neurons of mouse hippocampus, is the drop in the expression of the P2X_2_ receptor after the seizure (kainic acid model). Expression of remaining receptors (P2X_1_, P2X_3_, P2X_4_, P2X_5_) did not undergo any changes [19]. Activation of the P2X_2_ receptor promotes inhibitory neurotransmission in the hippocampus. In relation to the above, there are circumstances that might indicate whether its agonists may be used in epileptic therapy [17]. Another important type of receptor is P2X_4_. Studies assessing the expression of P2 receptors on microglial cells from mice hippocampus, taking place after the occurrence of status epilepticus, revealed an increased expression after 24 and 48 h in case of P2X_4_ receptors (a decrease was reported after 3 h, but it was not statistically significant), as well as P2X_1_, P2X_7_, P2Y_12_ and P2Y_6_ in particular (the most significant growth). Due to increased expression of P2Y_6_ and P2Y_12_ receptors, as well as due to their activation on microglial surface, it is possible that these cells will more quickly respond to damage with participation of “eat me” and “find me” signals mediated by extracellular nucleotides [23]. However, the activation of P2X_4_ receptors on the surface of microglia originating from the hippocampus influences its activity and contributes to the death of neurons, although it does not exert any influence on the course of seizures [24].

As far as studies focus on the role of agonists/antagonists of P2 receptors in pathophysiology of epilepsy are concerned, there are elaborations describing opposite, adverse activity or lack of such activity when compared with the above-mentioned, promising studies. These differences can result from specificity and the dose of administered substance, utilized model (including the manner of evoking epilepsy, e.g., by means of pilocarpine, quinolinic acid or picrotoxin), genetic tools, type of examined cells (neurons/microglia/astrocytes) or presence of acidosis (it inhibits P2X_7_ receptors), which is frequently associated with status epilepticus. It is essential to conduct further studies in order to address these doubts [17]. Despite this, it seems that using antagonists of receptors for nucleotides provides hope for substantial progress in epileptic treatment.

### 2.2. Pain—Physiological and Pathophysiological Mechanisms

ATP is considered to be one of the most essential transmitters participating in transmission of sensory stimuli, including painful stimuli, from peripheral nerves to the central nervous system (CNS), as well as in communication between immune system cells and nervous system cells. It is released from inflamed or damaged tissues and during synaptic transmission [20]. P2 receptors participate in ATP-mediated transmission of pain signals [7]. For example homomeric P2X_3_ and heteromeric P2X_2/3_ are expressed in afferent sensory neurons (mainly small-sized neurons, but also in medium- and large-sized neurons [21]), especially in neurons of the dorsal root ganglia and neurons of the trigeminal nerve participating in transmission of nociceptive stimuli, as well as the inferior ganglion of the vagus nerve and inferior ganglion of the glossopharyngeal nerve. They are also present in sensory nerve endings where are responsible for the pain sensation and temperature sensitivity. In cases where they are located in supraspinal structures they inhibit the transmission of painful stimuli. P2X_3_ receptor often (73%–84%) reveals a common expression with the P2Y_1_ receptor [91]. P2X_3_ and P2X_2/3_ receptors are also involved in initiation of visceral pain (*i.e.*, from the bladder, ureters, uterus, intestines and gallbladder) [91,92]. However, apart from these, other P2X and P2Y receptors exhibit expression in sensory neurons as well, and they are engaged in pain signal transduction. Expression of P2X_1_ and P2X_4_ receptors was observed in rat trigeminal ganglion neurons [93]. Another study conducted on rats revealed the expression of mRNA and proteins of P2Y_1_, P2Y_2_, P2Y_4_, P2Y_6_ receptors in dorsal root ganglia, trigeminal nerve and inferior vagus nerve [91]. This indicates that apart from the ATP, there are other nucleotides that can play the role of transmitters in this process, e.g., ADP. Malin *et al.* [94] showed the expression of P2Y_1_, P2Y_12_, P2Y_13_ and P2Y_14_ receptors in neurons of mouse dorsal root ganglia. Depending on the subtype of the receptor, they act pronociceptively (P2Y_1_) or antinociceptively (P2Y_12_, P2Y_13_, P2Y_14_). In response to ADP, the P2Y_1_ receptor evokes pronociceptive activity, and what is more, it is required for the full expression of inflammatory hyperalgesia. G_αi_ proteins-coupled P2Y receptors cause an opposite effect. They inhibit the excitatory signalling in sensory neurons (they inhibit N-type Ca^2+^ channels and decrease glutamate release), whereas their expression is regulated in response to an inflammation. Under proper conditions, opposite effects resulting from the activity of these receptors are balanced and they constitute a mechanism regulating a response to painful stimuli. Damages caused by an inflammation lead to impairing this balance to the benefit of pronociceptive activity and they can be seen in the form of hyperalgesia.

Apart from the pain received by peripheral receptors, occurring as a physiological reaction to tissue damage, clinical practice has seen patients with neuropathic pain. Neuropathic pain is caused by damage or dysfunction within the peripheral and/or central nervous system and is of paroxysmal character that can gradually transform into a permanent condition. Its pathophysiology is still not fully understood, and treatment methods frequently remain insufficiently successful [95]. It is present in about 3% of people all over the world. Etiology of this condition is quite diverse, e.g., spinal cord injury and certain cases of strokes (leading to central pain), nerve damage, multiple sclerosis, neuropathies in the course of metabolic disorders (diabetes and uraemia), neuropathies in the course of viral infections (HIV, herpes zoster), neuropathy as a distant manifestation of a neoplasm, alcoholism and vitamin and mineral deficiency often associated with alcohol abuse (such as deficiency in vitamin B6, PP, magnesium and zinc), long-lasting drug therapy, Lyme disease and many others. The course of the neuropathic pain results in changes within synaptic connections and pain transmission on the level of the peripheral and central nervous system. The main location showing an inappropriate activity includes lamina I of the spinal dorsal horn. Mechanisms responsible for inhibiting the pain undergo suppression, whereas the ones that facilitate pain are increased, which leads to exaggerated response to painful stimuli. In this situation, the sensory output from spinal neurons is not transmitted under the influence of painful stimuli as it is the case in physiological conditions, but without the presence of stimuli or in response to stimuli of quite a different character. That is why patients with neuropathic pain also observe symptoms such as allodynia (the sensation of pain under the influence of stimuli, which do not cause similar outcomes in healthy individuals), hyperalgesia (hypersensitivity to pain) or spontaneous pain (experiencing pain without any palpable reason) [96].

The development of neuropathic pain is associated with improper signalling between neurons. It was stated that P2X_3_ and P2X_2/3_ receptors located in sensory neurons play a crucial role in developing a chronic inflammatory pain, neuropathic pain [20], acute pain, migraine and cancer pain [91]; particularly these receptors, which are present in endings of primary afferent fibres in inner lamina II of the spinal cord (P2X_3_ receptor facilitates glutamate release) [20] and in the trigeminal brainstem sensory nuclei [28]. Their activation causes an increased sensitivity to painful stimuli [20] and it potentiates acute pain, as well as such symptoms as hyperalgesia and allodynia [22]. It has been stated that sensitisation of P2X_3_ receptors rather than the change in ATP release is responsible for neuropathic pain and allodynia [91]. The neuropathic pain occurs after peripheral nerve injury with concomitant tactile allodynia depends on the activation of cPLA_2_ (cytoplasmic PLA_2_) in primary sensory neurons [21]. The activity revealed by numerous antagonists of these receptors leads to decreased pain and sensitivity to painful stimuli, whereas the application of antisense oligonucleotides results in reducing the expression of the receptors and reduces mechanical hyperalgesia [91]. In case of neuropathic pain occurring after peripheral nerve injury, it seems possible to use agents acting on the level of cPLA_2_ [21]. Li *et al.* [78] report that yet another subtype of receptor—P2Y_2_, which is located in trigeminal ganglion neurons, may participate in initiating and maintaining the allodynia, and administering its antagonist reverses this activity.

What is important is the fact that not only dysfunctional neurons, as it has been previously assumed, but also damaged glial cells are responsible for the development of chronic pain. Numerous studies indicate that the main type of glial cells participating in neuropathic pain pathogenesis includes microglia, which influences neighbouring cells, for example neurons, by secreting bioactive factors. Purinergic signalling is of considerable significance in this process [25,88,97]. P2X_4_, P2X_7_ and P2Y_12_ receptors in spinal microglia are indicated as playing a certain role in developing neuropathic pain [92,96,98]. The P2X_4_ receptor is assumed to be one of the most important elements in neuropathic pain pathogenesis. It was noted that peripheral nerve injury causes an increased expression of P2X_4_ receptors in spinal microglia in a mechanism depending on IFN-β (interferon β)_._ These receptors mediate development of allodynia [25] and activate microglia (IFN-γ (interferon γ) is the notable factor in this process) [26]. P2X_4_ receptors are up-regulated in spinal microglia in response to nerve injury (but not in response to peripheral tissue inflammation) at the transcriptional, translational and post-translational levels. Factors regulating P2X_4_ expression on the transcriptional and translational level include CCL21 (chemokine (C-C motif) ligand 21), fibronectin in spinal cord (through Lyn kinase) and IRF8 (interferon regulatory factor 8), whilst on the post-translational level it is CCR 2 (C-C chemokine receptor type 2) [27]. Activating P2X_4_ receptor leads to release of BDNF (brain-derived neurotrophic factor) that mediates communication between microglia and neurons, and it leads to the occurrence of pain hypersensitivity [28]. BDNF alters the output of spinal lamina I neurons to the brain. It down-regulates the expression of K^+^-Cl^−^ co-transporter KCC2 (potassium chloride co‑transporter 2), the main Cl^−^ transporter in these structures, which causes changes in a considerable number of signals in neurons, from inhibitory to excitatory. The reason is the shift of the Cl^−^ reversal potential leading to a decreased inhibition of the excitatory transmission by GABA_A_ receptors. On the other hand, blocking the P2X_4_ receptor results in reversing the allodynia [96]. What is more, these symptoms were also not observed in P2X_4_-deficient mice [29].

P2X_7_ receptor is crucial in pathogenesis of the inflammatory pain (chronic condition) or pain after nerve injury [38,92]. Participation of this receptor is confirmed by works that observed: (1) an increased expression of this receptor in microglia after injuring peripheral nerves; (2) lack or reduced neuropathic pain symptoms after using its antagonists, as well as in P2X_7_-deficient animals [38,39,40]. Apart from the above, the IL-1β released by microglia depending on the P2X_7_ receptor, has the capacity to alter the pain sensitivity. Another mechanism that is important for neuropathic pain to occur and that depends on the P2X_7_ receptor is the change in glutamate release by neurons and astrocytes [38].

An increased expression (both mRNA and protein of the receptor) of P2Y_12_ receptor on the surface of microglial cells was also observed in response to peripheral nerve injury, and the use of its specific antagonists prevented the occurrence of allodynia [88]. Described activity of microglial P2Y_12_ receptor is contradictory to the one described when it is located in neurons of dorsal root ganglia where it exhibits an antinociceptive activity. Within this context it would be advised to conduct further studies on the harmfulness associated with using this receptor antagonist in persistent pain that lacks a significant contribution from microglia [94]. Other subtypes of P2Y receptors that are suspected to play a noteworthy role in neuropathic pain are P2Y_6_, P2Y_11_, P2Y_13_ and P2Y_14_. Barragan-Iglesias *et al.* [85] showed that P2Y_6_ and P2Y_11_ receptors show expression in spinal microglia and they are up‑regulated in response to spinal nerve injury. Administration of selective antagonists of both receptors reduced tactile allodynia and spinal nerve injury-induced increase in receptor expression. This was confirmed by Kobayashi *et al*. [86]. They observed an increased expression of P2Y_6_, P2Y_13_ and P2Y_14_ in spinal microglia, and reduced mechanical pain hypersensitivity after administration of antagonists of these receptors. Peripheral nerve injury also resulted in increased expression of P2Y_6_ receptor in mice primary afferent neurons, yet administering an antagonist did not influence the injury-induced neuropathic pain behaviour [99].

Summing up, when ATP stimulates purinergic receptors, it may modulate nociceptive sensitivity by direct influence on neurons (P2X_3_, P2X_2/3_ and P2Y_2_) or indirectly by interacting between microglia cells and neurons (P2X_4_, P2X_7_ and P2Y_6_, P2Y_11_, P2Y_12_, P2Y_13_ and P2Y_14_) (Table 1). This is of considerable significance as far as neuropathic pain pathogenesis is concerned. Interestingly, P2X_4_, P2X_7_ and P2Y_12_ receptors use the same signalling pathway—p38-MAPK (p38-mitogen-activated protein kinase)—which indicates that there is a point of convergence in pathogenesis of the neuropathic pain. The significance of this convergence is unclear and requires elucidation [96]. Selective antagonists may probably be used in new therapeutic methods.

### 2.3. Depression

Depression is a mental disorder, with low mood and reduced psychomotor drive, circadian rhythm abnormalities and anxiety disorders. About 10% of population suffer from this condition, which has a tendency to recur and is associated with suicidal ideation. A widely known etiologic theory of depression associates with deficiency in biogenic monoamines, namely norepinephrine and serotonin. All available antidepressants act, at least in part, by increasing monoaminergic transmission [100]. According to the current state of knowledge, we know that depression is also associated with a chronic, low-grade inflammatory response, activation of cell-mediated immunity and CIRS (compensatory anti-inflammatory reflex system). The presence of an acute inflammation is as well risk factor for the development of *de novo* depression [101]. In relation to the above, the prevalence of depression is high among patients suffering from conditions with concomitant inflammation, including neurodegenerative, autoimmune and communicable diseases [7]. It is currently assumed that the occurrence of mood disorders depends on interaction between genetic, developmental and environmental factors [102].

Numerous studies indicate that proinflammatory cytokines (IL-1β, IL-6, TNF-α, IFN-α (interferon α), IFN-γ, soluble receptor for IL-6, antagonist of the receptor for IL-1) are crucial factors in etiopathogenesis of depression [100,101]. It has been stated that an increased microglial density and a higher level of proinflammatory cytokines can be observed in patients suffering from depression but not experiencing any concomitant diseases [7]. Proinflammatory cytokines may induce stress reactive neuroendocrine and central neurotransmitter changes reminiscent of those in depression [100]. In relation to low efficiency and associated side effects, it is essential to create new antidepressants, which could decrease the inflammation and would regulate the level of biogenic amines, based on another mechanism of action. Utilising antagonists of purinergic receptors seems highly promising.

The P2X_7_ receptor can be of great significance in the context of depression. Selected SNPs (single nucleotide polymorphisms) in the *P2X7* gene are responsible for increased susceptibility to BDP (bipolar disorder) and MDD (major depressive disorder), and the most commonly studied polymorphisms are Gln460Arg (rs2230912) and His155Tyr (rs208294) [7,41,42,43,103]. These polymorphisms may affect the function of P2X_7_ receptors through changing the subunit interaction, agonist binding and/or channel gating [102]. Mantere *et al.* [44] indicated that His155Tyr polymorphism is associated with higher neuroticism among patients, which in turn predicted a higher proportion of time spent in mood episodes. Subsequent studies, did not indicate differences within the frequency of Gln460Arg polymorphism between patients suffering from BDP and MDD and control, however, researchers observed its association with severity of depression symptoms [45,46]. Viikki *et al.* [104] obtained similar study results, as they declared that Gln460Arg and His155Tyr polymorphisms are not related with MDD and remission after selective serotonin reuptake inhibitors or electroconvulsive therapy. Mechanisms responsible for the occurrence of changes in behaviour under the influence of *P2X7* gene polymorphisms is not fully explained and requires further research.

The role played by P2X_7_ receptors in depression development was also studied on animal models. Basso *et al*. [47] found that P2X_7_ KO mice exhibit an antidepressant-like profile in tail suspension test and forced swim test, most probably in connection with inhibition of IL-1β release and high sensitivity to antidepressants (imipramine), whereas antagonists of P2X_7_ receptor could be potentially useful in the treatment of affective disorders. IL-1β inhibits the expression of BDNF in hippocampus [105], a factor that supports survival of existing neurons (including serotonergic neurons), stimulates growth and differentiation of new neurons, as well as creation of synapses, and also influences synaptic transmission [106]. Stokes *et al.* [48] confirm role of IL-1β, as they noted that the presence of high or gain-of-function SNPs in the *P2X7* gene, e.g., Gln460Arg, is associated with increased release of IL-1β by peripheral blood monocytes in response to ATP. Csolle *et al.* [49] contradict the role attributed to IL-1β derived from haematopoietic cells, such as monocytes or microglia, as a factor responsible for mood disorders depending on P2X_7_, although they do not exclude the participation of other nervous tissue cells, e.g., astrocytes, in this process. They also observed a mood-stabilizing phenotype in several behavioural models in the absence of P2X_7_ receptors on non-haematopoietic cells (but not in case of haematopoietic cells), as well as after administering BBG (Brilliant Blue G). Currently, more and more focus is devoted to the role of glutamatergic signalling in depression pathogenesis. In their subsequent work they indicate participation of several potential mechanisms for the antidepressant phenotype of P2X_7_^−/−^ mice, namely the absence of P2X_7_-mediated glutamate release, elevated basal BDNF production, enhanced neurogenesis in dentate gyrus, as well as increase of 5-HT bioavailability in hippocampus [50]. P2X_7_ receptor and ATP are responsible for increased glutamate release from presynaptic endings, and what is more, they also influence the number of AMPA receptors (α-amino-3-hydroxy-5-methyl-4-isoxazolepropionic acid receptors) in presynaptic membrane, that result in excessive synaptic transmission and can contribute to the development of depression disorders [51].

Available data based on analysis referring to SNPs and animal models indicate the role of the P2X_7_ receptor in depression pathogenesis (Table 1). Its influence most probably results from the ATP-dependent release of cytokines (suggested role of IL-1β) by microglial cells or other cells (monocytes, astrocytes), which finally leads to deficiency in biogenic monoamines. Antagonists of this receptor exhibit anti-depressive activity in animals, and seem to be useful in the treatment of mood disorders.

### 2.4. Neurodegenerative Diseases

Neurodegenerative diseases include a wide group of congenital or acquired nervous system conditions, which are characterized by progressive loss of neurons and leads to motor (ataxia) and memory impairment (dementia). These diseases include Alzheimer’s disease, Parkinson’s disease, multiple sclerosis, amyotrophic lateral sclerosis, Huntington’s disease and many other, less renowned diseases. What is currently being emphasized is the participation of neuroinflammation, as microglial cells play an important role in this process, in pathophysiology and progression of neurodegeneration. Numerous works emphasise the significance of purinergic signalling in induction of inflammatory response and neurodegenerative conditions. Both neurons and glial cells exhibit an expression of P2X and P2Y receptors. ATP, as well as other nucleotides released from neurons and glial cells in the course of neurodegeneration and neuroinflammation, plays the role of signalling molecules that can participate in the development of pathological changes [107].

#### 2.4.1. Alzheimer’s Disease (AD)

Alzheimer’s disease is a neurodegenerative disease that is the most common cause of dementia. This disease is associated with global cognitive decline including a progressive loss of memory, orientation and reasoning. It currently remains incurable. At present, the cause of AD is not well understood, but it is related to progressing death of neurons and loss of synapses (cerebral cortex and certain subcortical areas), which results from accumulation of pathological protein deposits in the brain—namely β-amyloid forming senile plaques and hyperphosphorylated tau protein forming neurofibrillary tangles [107]. Among causes of senile plaques formation, researchers enumerate the decreased rate of microglial β-amyloid clearance [79] and chronic activation of these cells [108]. Neurotoxic β-amyloid is a product resulting from the activity of β-secretase and γ-secretase, which cleave the APP (amyloid precursor protein) [52]. β-amyloid induces free-radical reactions and inflammation, and it finally leads to death of neurons and development of dementia. On the other hand, the activity of α-secretase leads to the creation of soluble sAPP-α (secreted amyloid precursor protein-α) [109], which is characterized by neuroprotective and neurotrophic properties [110].

Numerous studies are devoted to the significance of purinergic signalling in AD development (Table 1). What remains crucial is the fact that neurons damaged in the course of the disease release considerable amounts of nucleotides, which participate both in its progression, as well as in inducing neuroprotective mechanisms. Released ATP may act as a “find me” signal for microglial cells [111], whilst ATP and UTP induce astrocytes migration [112]. These cells accumulate around β-amyloid deposits in the brain [107,113]. Astrocytes, which were activated and accumulate around amyloid plaques are probably responsible for β-amyloid homeostasis and modulation of the neuronal environment [113]. β-amyloid is one of the factors that induce ATP release by astrocytes [114] and microglia [79]. Extracellular nucleotides take part in the development of inflammation by inducing the microglial chemotaxis [115], proliferation, cytokines release (IL-1β, IL-6, TNF-α), phagocytosis [116], adhesion [117] and astrogliosis [10] in response to damaging factors. Astrogliosis is a well described AD feature [110]. Accumulation of proinflammatory cytokines as well as β-amyloid contributes to progression of AD [118].

Inflammation constitutes an essential element of neurodegenerative diseases, including AD. In the initial stage it plays the role of neuroprotective mechanism by secreting cytokines, growth factor, chemokines and clearing neurotoxic β-amyloid from tissues [114]. Microglial cells are responsible for β-amyloid internalisation and degradation, as well as for production of chemotactic factors, such as MCP-1 and IL-1β [79]. The inflammation becomes the neurodegenerative mechanism when its duration is prolonged [110]. Chronic inflammation results from maintaining activation of astrocytes and microglia in response to oxidative stress, which is postulated as an important factor in the initial stage of AD. Oxidative stress also enhances the production of β-amyloid [114]. Activation of microglial P2X_7_ receptors by ATP or BzATP stimulates an increased production of superoxide anion radicals (O_2_^−^), which induce cell death in cortical neurons [53]. According to Kim *et al.* [119] in this particular case microglial cells stimulated by β-amyloid constitute the source of ATP. These researchers confirm that this mechanism is involved in activating an NADPH oxidase and in inducing oxidative stress.

Presence of various inflammatory mediators was reported in patients with Alzheimer’s diseases, including cytokines, chemokines, proteases and protease inhibitors. Microglia participate in AD progression by releasing proinflammatory cytokines of neurotoxic activity. IL-1β [80] is one of these cytokines and microglia are its main source (astrocytes release it in smaller amounts) [120]. It is responsible for regulating the expression of many proinflammatory mediators in the AD brain [118], for increasing the expression and processing of APP, as well as for phosphorylating tau protein, which may contribute to damaging neurons and microglial activation [80]. Just like TNF-α and ATP, IL-1β is released by P2X_7_ receptor-activated microglia [118,121]. In response to β-amyloid stimulation, release of IL-1β, but not TNF-α, may be modulated by extracellular ATP [120,122]. According to Bernardino *et al.* [54] P2X_7_ receptor-dependent release of IL-1β may prime neuronal susceptibility to a subsequent excitotoxic insult, and it is mandatory for exacerbation of neuronal loss. An increased expression of P2X_7_ receptor in microglia located around β-amyloid deposits was noted in the mice AD model [53]. This expression was also increased in adult human microglia with AD, as well as fetal human microglia after exposure to β-amyloid [55]. Properties of the P2X_7_ receptor may be used in novel therapies, where administration of proper antagonists may prevent adverse effects resulting from chronic neuroinflammation, as well as pathological activity in glial cells [56]. Ryu and McLarnon [57] observed a neuroprotective activity and an inhibiting inflammatory response after administering one of P2X_7_ receptor’s antagonists. Administration of BBG led to reduced expression of this receptor on the surface of rat neurons, attenuated gliosis and diminished leakiness in the brain-blood barrier, which was induced by previous administration of β‑amyloid.

β-amyloid also induces neuronal dysfunction and death by disruption of Ca^2+^ homeostasis. It is believed that it evokes the above conditions by an increased Ca^2+^ flux through NMDA (*N*-methyl-d-aspartate receptor) and VOCC (voltage-operated calcium channel) channels, however, using their antagonists/modulators have shown limited efficacy in clinical trials to treat AD. According to Varma *et al.* [30] β-amyloid induces accumulation of Ca^2+^ permeable P2X_4_ receptors on the surface of neurons, as well as induces their caspase-3-mediated proteolytic processing. They all lead to slowed closure times of these channels and prevented agonist-induced internalisation of the receptors, which finally leads to increased Ca^2+^ flux.

Kong *et al.* [80] observed an increased expression of P2Y_2_ receptor and its activity in rat cortical neurons under the influence of IL-1β. Under physiological conditions, the P2Y_2_ receptor exhibits low expression and is unresponsive to UTP [114]. UTP activated this receptor, which resulted in sAPP-α release in a mechanism depending on ADAM10/17 (disintegrin and metalloproteinase domain-containing protein 10/17) and PI3K (phosphoinositide 3-kinase) activity, whereas ERK1/2 (extracellular signal-regulated kinase 1/2) and PI3K activity may indirectly regulate APP processing [80]. Neuronal P2Y_2_ receptors also participate in regulating phosphorylation of one cytoskeletal protein—cofilin, which finally leads to stabilising the outgrowth of neurites and dendritic spines. This is yet another neuroprotective mechanism in the course of AD that involves participation of this receptor [110]. Human cell 1321N1 astrocytomas also exhibit the release of sAPP-α after activating P2Y_2_ receptors (UTP), depending on the activity of ADAM10 and ADAM17/TACE (disintegrin and metalloproteinase domain-containing protein 17/tumour necrosis factor-α-converting enzyme) [81]. The process of P2Y_2_ receptor-dependent activation of α-secretase and sAPP-α release prevents the formation of β-amyloid from the same APP molecule [114]. Delarasse *et al.* [52] stated that besides the P2Y_2_ receptor, also the previously mentioned P2X_7_ receptor induces sAPP-α production and release (in human and mice neuroblastoma cells, as well as mice primary astrocytes and progenitor neural cells). This process depends on ERK1/2 and JNK (c-Jun *N*-terminal kinase) phosphorylation, which leads to activation other than the previously described metalloprotease for sAPPα release. According to Tran, rat cortical astrocytes are the source of APP, and its production and release may be regulated by activation of P2Y_2_ and P2Y_4_ receptors coupled to MAPK pathway [82].

Expression of P2Y_2_ receptors under the influence of β-amyloid (most probably with participation of IL-1β) was also increased in mice microglia, which revealed increased mobility and capacity to uptake and degrade this protein in response to ATP or UTP [79]. This is in compliance with other studies conducted on the mouse model, where P2Y_2_ receptors are crucial for proper microglial recruitment and activation, as well as induction of β-amyloid clearance by these cells. Reduced expression among these receptors was associated with shorter survival time and more rapid onset of neurological deficits [83]. Post-mortem evaluation of samples from AD patients revealed a decreased expression of P2Y_2_ receptor in brain regions that are typical for AD, when compared with healthy controls, whereas expression of P2Y_4_ and P2Y_6_ receptors remained unchanged [84]. This indicates that P2Y_2_ receptors may take part in regulating neuroprotective mechanisms and they constitute a potential aim for studies on agents, which will prevent death of neurons in the course of AD.

Concluding, the β-amyloid present in the course of Alzheimer’s disease as well as the chronic stress and neuronal injury induces release of considerable amounts of nucleotides. This leads to induction of the inflammatory response in glial cells and to release of proinflammatory cytokines such as IL-1β and more nucleotides, which may cause the following: (1) progression of the disease by inducing oxidative stress and by enhancing the accumulation of β-amyloid and tau protein deposits; (2) neuroprotective activity by increasing the expression of neuronal P2Y_2_ receptor by means of IL-1β, whose activation results in production of soluble sAPP-α and in rearrangement of the neuronal cytoskeleton; the P2Y_2_ receptor is also responsible for mobilising microglia to β-amyloid deposits clearance. ATP is probably the crucial inflammatory mediator in the course of AD. The meaning of ATP as a factor inducing both neurodegenerative, as well as neuroprotective mechanisms requires explanation.

#### 2.4.2. Parkinson’s Disease (PD)

Parkinson’s disease is a neurodegenerative disease of the CNS, characterized by a slow, progressing course and currently remains an incurable condition. During the course of the disease, dopaminergic neurons located in the substantia nigra die. This results in dopamine deficiency. Therefore patients with PD suffer from impaired motor skills as well as mood, cognitive, speech, sensation and sleep disturbances [107]. Neuroinflammation is currently assumed to be the main factor in PD pathogenesis [58]. Purinergic signalling is enumerated among significant processes as far as this disorder is concerned, mainly due to the role it plays in inducing the neuroinflammation, although at present the amount of data is limited (Table 1).

Receptors from the P2 family are observed not only in healthy, but also in damaged nigrostriatal system. Dopaminergic neurons in the substantia nigra pars compacta in an adult rat express P2X (P2X_1_, P2X_2_, P2X_3_, P2X_4_, P2X_5_, P2X_6_) and P2Y (P2Y_1_, P2Y_4_, P2Y_6_, P2Y_14_) receptors. Astrocytes within this region express the P2Y_2_ receptor, whereas microglial cells express the P2X_7_ receptor. Administration of 6-OHDA (6-hydroxydopamine) (unilateral nigral 6-OHDA rat model of PD) resulted in the loss of dopaminergic neurons and considerable decreased expression of the above-mentioned receptors. Interestingly, GABAergic neurons (P2X_1_, P2X_3_, P2X_4_, P2X_6_ receptors) and astrocytes (P2Y_4_ receptor) from substantia nigra pars reticulate are characterized by higher expression of purinergic receptors, and this mechanism may compensate the dopamine deficiency [123].

In PD, as in the case of AD, neuroinflammation also plays a crucial role in development of this disorder. Microglial cells are chronically stimulated by substances released from dead neurons and activated astrocytes, including extracellular nucleotides [124]. Released ATP may act as a factor accelerating the progression of the PD caused by genetic or environmental factors [58]. Activated microglia of the substantia nigra releases ROS and cytokines such as IL-1β, IL-6 and TNF-α, which during the initial stage may play the protective role towards neurons, however, as the disease progresses, these may become neurotoxic and may exacerbate the neurodegeneration [124,125]. The following was noted in dopaminergic nigrostriatal region and in cerebrospinal fluid of PD patients: (1) increased level of proinflammatory cytokines such as IL-1β, IL-2, IL-4, IL-6 and TNF-α; (2) increased level of factors associated with apoptosis, including TNF-α receptor R1 (p55), soluble Fas, Bcl-2 and elevated caspase-1 and caspase-3 activities; (3) lowered level of neurotrophins, such as BDNF and NGF (nerve growth factor) [126].

P2X_7_ is the main P2 receptor evaluated in PD. Studies on a Chinese population revealed an association between certain SNPs in the *P2X_7_* gene and the risk of sporadic PD, late-onset PD and male PD [59]. Marcellino *et al.* [60] report that P2X_7_ receptors of substantia nigra are mainly located on microglial cells and on astrocytes. However, the presence of the P2X_7_ receptors in dopaminergic neurons was not confirmed. Activation of P2X_7_ receptors on microglial cells leads to secretion of proinflammatory cytokines, such as IL-1β, and it induces neuroinflammation. This is how P2X_7_ receptor can participate in neurodegeneration within the substantia nigra or it may influence neuronal function in this specific structure. Blockade of the P2X_7_ receptor-induced microglial activity constitutes an attractive neuroprotective strategy for patients with PD.

Astrocytes are another population of nerve tissue cells that are known for their crucial role in neuroinflammation and neurodegeneration. Post-mortem study performed in patients with PD (substantia nigra) revealed a mild or moderate reactive astrogliosis [124]. Induction of astrogliosis, inflammation, apoptosis/necrosis, cytokine and chemokine release occurs with the participation of multiple astrocytal P2 receptor family members [113]. As mentioned above, astrocytes of substantia nigra in rats express P2X_7_ receptors [60], as well as P2Y_2_ (pars compacta) and P2Y_4_ (pars reticulata), and their expression levels change when PD is induced by 6-OHDA [123]. Basing on a PD rotenone model Gao *et al.* [124] revealed that the expression level of P2X_7_ receptor in midbrain astrocytes after rotenone administration does not change, but what can be seen is an increase in receptor current density, as well as extended channel deactivation time, and thereby inhibition of TNF-α secretion. Administration of antagonist of P2X_7_ receptor, BBG, reverses this effect, and even promotes the secretion of TNF-α.

Jun *et al.* [58] reported involvement of neuronal P2X_7_ receptor in PD. They stated an expression of these receptors in SN4741 dopaminergic neurons derived from the substantia nigra of transgenic mouse embryos. ATP induced swelling and cell death. It probably has a necrotic (features of necrosis), and not apoptotic, character. What is also possible is the fact that other receptors that reveal an expression on these cells, for example P2X_2_ and P2X_4_, also may induce such activity, although to a much smaller extent. Previously mentioned studies [60,123] indicate the lack of P2X_7_ receptors expression in adult dopaminergic neurons, which was confirmed by Heine *et al*. [127]. These researchers suggest a potential role of this receptor in the neuronal development and growth, which would explain their presence in neurons obtained from embryos. What is more, P2X_7_ receptor participates in pathogenesis of PD by having toxic effect on striatal synaptosomes and neurons (SH-SY5Y neuroblastoma cells) and induction of gliosis [61].

Studies with the use of P2X_7_ receptor antagonists are being conducted. Marcellino *et al.* [60] stated that administration of A-438079, partially, but significantly prevents the 6-OHDA-induced depletion of striatal dopamine stores. Nevertheless, it does not reduce the loss of neurons in the substantia nigra. BBG attenuates the 6-OHDA-induced: increase of contralateral rotations in the apomorphine test, short-term memory impairment, reduction of dopamine stores in substantia nigra and striatum, as well as microgliosis and astrogliosis in the striatum. BBG also prevented dysfunctions of striatal synaptosomes and neurotoxicity [61]. Results of studies focusing on P1A_2A_ receptor antagonists, which may have protective activity for dopaminergic neurons are also very promising [128].

Analysis of the above-mentioned works indicates that microglial P2X_7_ receptors play a crucial role in PD development and progression, as these receptors induce IL-1β-related neuroinflammation, and by the same they contribute to the occurrence of neurodegenerative lesions in substantia nigra and to dopamine deficiency. Administration of P2X_7_ receptor antagonists prevents the development of excessive inflammation, neuronal damage and PD symptoms evoked in animal models.

#### 2.4.3. Multiple Sclerosis (MS)

Multiple sclerosis is a disease that usually begins with an acute autoimmune inflammatory reaction to myelin components of the CNS. Progression to a chronic phase leads to degeneration of oligodendrocytes, myelin and axons, which is related to cytotoxic activity of substances released by immune cells, as well as excitotoxicity, and finally results in neuron damage and demyelination. These pathological changes cause a series of both physical and mental symptoms, the progression of which lead to physical and cognitive disability. The white matter is indicated as the main area where the disease process is taking place, however, presence of pathological lesions was also noted in grey matter (brain cortex). Astrocytes and microglial cells are engaged in the pathogenesis of MS. Available studies suggest that their activity may be mediated by extracellular nucleotides (Table 1) [89,107].

In the course of MS, activated immune system cells, dead oligodendrocytes and neurons, as well as activated astrocytes release considerable amounts of nucleotides, which may evoke excitotoxic degeneration of cells [62]. ATP is released via pannexin1 (Panx1)/P2X_7_ receptor channels. Activation of P2X_7_ receptors opens Panx1 channels, which become permeable for ATP (a pro-inflammatory factor) and Ca^2+^ (inflow into cells) and hence contribute to cellular death. This mechanism is engaged in developing EAE (chronic experimental autoimmune encephalomyelitis; animal SM model) in the spinal cord. In Panx1 knockout (KO) mice, ATP release was decreased and an up-regulation in the P2X_7_ receptor was seen (perhaps as a compensatory mechanism). Clinical EAE symptoms occur with a certain delay, inflammatory lesions in the spinal cord observed during the acute phase of EAE and the mortality were reduced. Pharmacological inhibition of Panx1 attenuates the course of the disease [63].

Rat OPCs (oligodendrocyte progenitor cells) express several subtypes of P2 receptors: P2X_1_, P2X_2_, P2X_3_, P2X_4_, P2X_7_ and P2Y_1_, P2Y_2_, P2Y_4_. P2X_7_ and P2Y_1_ receptors are the main active receptors. In physiological conditions, axons that generate action potential release ATP, which undergoes degradation to ADP and through P2Y_1_ receptors, it inhibits their proliferation and stimulates migration and differentiation [11,129]. Expression of the P2X_7_ [62] and P2Y_12_ [89] receptor is detected on the surface of differentiated oligodendrocytes. Depending on the activity of neurons and environmental factors, mature oligodendrocytes may be mobilized to myelinate (remyelination). ATP released by axons stimulates LIF (leukemia inhibitory factor) release by astrocytes, which stimulates myelination on subsequent stages of development. It is not clear which subtypes of P2 receptors are responsible for this particular activity. High LIF (or ATP) concentration may, however, contribute to inhibiting myelination or remyelination, which is observed in the course of demyelination diseases. These properties constitute a promising target for MS therapy [130].

Numerous processes reported in the course of MS are mediated with participation of the P2X_7_ receptor. Patients with MS show increased expression of P2X_7_ receptor in normal-appearing axon tracts [62], reactive astrocytes [131] and neurons and astrocytes during an acute EAE phase [63,64], which suggests their participation in the development of the disease. By activating astrocytal P2X_7_^−^ receptors, ATP stimulates glutamate release, which is said to be excitotoxic for oligodendrocytes. Microglial cells, which release IL-1β in response to ATP, may exacerbate this process, as IL-1β increases the expression of P2X_7_ receptor on the astrocytal surface. ATP released in significant amounts may also chronically activate P2X_7_ receptors on the surface of oligodendrocytes, leading to their death. These processes result in lesions characteristic for MS, including demyelination, death of oligodendrocytes as well as axonal injury. Based on the animal model of MS, antagonists of the P2X_7_ receptor prevent demyelination and attenuate the functional decline resulting from the progression of the disease and most probably relieve the EAE symptoms [62]. On the other hand, it has been suggested that the P2X_7_ receptor is crucial for proper functioning of lymphocytes. P2X_7_^−/−^ mice revealed an enhanced susceptibility to EAE compared to wild type mice. Chen and Brosnan [65] explain this with the loss of P2X_7_ receptor-dependent apoptotic activity in lymphocytes. Contrary to previous studies, subsequent similar research by Sharp *et al.* [66] indicated a decreased incidence of EAE in P2X_7_^−^ null mice when compared to wild type mice. These differences may most likely result from varied techniques in the recombination of studied mice. Authors of the work excluded the fact that T lymphocyte activation or their ability to inflow into the CNS were factors initiating the development of the disease, and the reduction in axonal damage with decreased astrocytal activity suggest the role of astrocytal P2X_7_ receptors in EAE pathogenesis. This is also supported by results presented by Grygorowicz *et al.* [64], even though the participation of astrocytes in this process still requires confirmation. Apart from the above, certain SNPs in the *P2X_7_* gene predispose to MS [67]. This indicates the engagement of the P2X_7_ receptor in MS development and progression.

The P2X_4_ receptor is yet another receptor subtype from the P2 receptor family, which can be of considerable significance in MS. Its expression was revealed on the surface of macrophages infiltrating the rat CNS in the course of EAE, although not in normal, control rats. P2X_4_^+^ macrophage accumulation correlated with severity of the EAE [31]. Its increased expression was also observed in inflammatory foci, activated microglia in spinal cord of rats with EAE and the optic nerve of patients with MS [32]. Researchers suggest that this receptor plays a role in CNS inflammation [31], as well as in the control of the fate and survival of activated microglia [32].

Presence of the P2Y_12_ receptor was reported in oligodendrocytes, myelin and interlaminar astrocytes, where it can directly/indirectly participate in inducing myelination. Decreased expression of this receptor in the axon-myelin interface in white and grey matter in patients with MS correlated with increasing demyelination and overall reduction of MBP (myelin basic protein) in myelin sheaths and oligodendrocytes. Absent or reduced expression of P2Y_12_ receptors in interlaminar astrocytes is frequently observed among patients with neurodegenerative conditions [89]. Amadio *et al.* [90] suggest that changes in the expression of the P2Y_12_ receptor may play role as analytic marker of demyelinating lesions in the course of MS.

The above-mentioned reports indicate that nucleotide receptors, mainly P2X_4_ and P2X_7_, are engaged in the development of SM and they influence its course. Both subtypes of these receptors are responsible for inducing inflammatory response, and apart from the above, the P2X_7_ receptor exacerbates glutamate excitotoxicity and induces death of oligodendrocytes, simultaneously contributing to death of neurons and the occurrence of changes characteristic for SM. Studies with the use of antagonists for P2X_7_ receptors indicate that inhibiting these receptors may delay the disease progression and attenuate its symptoms.

### 2.5. Nervous System Neoplasms

#### 2.5.1. Glioma

Gliomas are a heterogeneous group of primary CNS neoplasms (brain and spinal cord tumours) originating from glial cells. These neoplasms constitute about 80% from among all diagnosed malignant brain tumours [132]. These are characterized by an extreme invasiveness, which contributes to high mortality in the group of patients with this tumour, despite surgery and adjuvant conventional radio- and chemotherapy [133,134,135]. Mechanisms responsible for transforming proper glial cells into malignant glioma cells are poorly known. Nonetheless, interactions between glioma cells and the tissue microenvironment, including neighbouring cells (neurons, glial cells and vessels), is essential. Vascular endothelial growth factor (VEGF) is vitally important as far as neoangiogenesis is concerned, as it is the growth factor secreted by human glioma and C6 rat glioma cell line [136]. The process of neoplasia also engages purinergic signalling (Table 1) [137], which participates in regulating proliferation, differentiation, mobility and cell death [138]. The elucidation of the mechanisms responsible for the tumour growth and invasiveness, as well as angiogenesis may enable the development of new therapies.

Glioma cells have the capacity not only to release ATP, but also to respond to this nucleotide [133], and what is more, they present a clear resistance to the death induced by concentrations of ATP, which are cytotoxic for normal cells [136,139]. It is being assumed that it acts as a signalling molecule in regulating the development of such neoplasms. Morrone *et al.* [140] stated that ATP and adenosine increase proliferation of various types of glioma cells. Inducing proliferation results from activating several intracellular pathways. ATP and adenosine stimulate ERK phosphorylation, PCK and PI3K/Akt activation in U138-MG human glioma cells [135]. Expression of P2Y_1_, P2Y_2_ and P2Y_12_ receptors on the surface of the C6 glioma cell line was also detected. Activation of the P2Y_12_ receptor (high expression) induced by ADP or its synthetic agonists results in activating ERK 1/2 and PI3K/Akt, whereas activation concerning the P2Y_1_ receptor (low expression; high in normal cells) leads to inhibition of the Akt pathway. High expression of the P2Y_12_ receptor and low expression of the P2Y_1_ receptor favours inducing the growth and proliferation of glioma cells, as well as inhibiting their differentiation. Apart from the above, the occurrence of such receptor expression profile is enumerated among mechanisms enabling survival of C6 glioma cells in unfavourable metabolic conditions [76].

Jantaratnotai *et al.* [133] demonstrated that stimulating the C6 glioma cell line by ATP leads to increased expression and production of the chemokines, IL-8 and MCP-1, as these recruit microglial cells and tumour-associated macrophages establishing the immunosuppressive microenvironment facilitating tumour growth. The described ATP effect depends on Ca^2+^ influx into cells through SOC (store-operated calcium channel), channels located in nonexcitable cells membrane that are activated in response to depletion of Ca^2+^ stores in the endoplasmic reticulum [141]. ATP most probably acts through metabotropic P2Y receptors, which mobilise intracellular Ca^2+^ through IP_3_ (inositol trisphosphate). This is not associated with changes in expression of these receptors (P2Y_1_, P2Y_2_, P2Y_4_, P2Y_6_) [133]. In their further studies, the same authors indicated that P2Y_1_ is the receptor engaged in this process [77]. Activation of the P2X_7_ receptor induces release of IL-8 and MCP-1 as well as increases VEGF and P2X_7_ receptor expression [68]. Administration of the P2X_7_ receptor antagonist BBG reduced tumour volume versus that in controls (intravenous administration; effect depending on the site of injection), as well as reduced migration of glioma cells and expression of this receptor induced by BzATP; however, BBG did not pose any influence on angiogenesis [69]. Depending on the dose, Fang *et al.* [70] observed an adverse effect of BBG—exacerbated tumour growth—through directly promoting cell proliferation and angiogenesis. EGFR, HIF-1α (hypoxia-inducible factor-1α) and VEGF receptors are probably engaged in this process, as their expression increases under the influence of BBG. Currently it is impossible to unambiguously determine the influence posed by activation/inhibition of P2X_7_ receptor on glioma growth, and to indicate mechanisms responsible for the above.

Ectonucleotidases (E-NTPDases (ectonucleoside triphosphate diphosphohydrolases), E-NPPs (ectonucleotide pyrophosphatasea/phosphodiesterases), ecto-5'-nucleotidase and alkaline phosphatase) are important elements in regulating the activity of nucleotides, which degrade extracellular nucleotides into di- and monophosphates and nucleosides. It has been stated that catabolism of adenine nucleotides in glioma cells is changed, which contributes to tumour growth and progression. When compared to astrocytes, they are characterized by decreased ATP hydrolysis (reduced activity of ATPases) and increased AMP hydrolysis (increased activity of AMPases) within the extracellular space, which leads to ATP and adenosine accumulation in the tumour environment [137]. Increased ATP concentration and increased concentration of glutamate released by tumour cells are cytotoxic for neighbouring cells (including neurons) and cause a release of significant amounts of both these substances from dead cells into the extracellular space, which results in the occurrence of positive feedback. The result of the above is associated with intensified proliferation of glioma cells and cytotoxic activity towards neurons and hence supporting to open space for rapid tumour growth [137,139].

Low expression of NTPDases responsible for ATP degradation in C6 glioma cells was observed, when compared with normal astrocytes [136,137,142]. Apyrase causes a reduction in the level of VEGF and angiogenesis, mitotic index and tumour size, in addition to its decreased invasiveness [136]. Braganhol *et al.* [142] studied the role of NTPDase2, the main E-NTPDase of astrocytes in culture, in tumour progression. In order to do so, expression of this enzyme was selectively re-established in C6 glioma cells. Its overexpression *in vivo* (but not *in vitro*) increased proliferation, angiogenesis, tumour growth, as well as platelet activation and their sizable sequestration in the tumour area. This effect may result from the fact that NTPDase2 degrades ATP to a greater extent than ADP, which leads to accumulating ADP, an agonist of platelet receptors, namely P2Y_1_ and P2Y_12_. Overexpression of NTPDase2 also contributes to increasing production of proinflammatory cytokines, IL-1β, TNF-α and IL-6, which intensify the progression and local invasion of tumour [137].

Ecto-5'-nucleotidase/CD73, which is responsible for degrading AMP to adenosine, is highly expressed in glioblastoma multiforme cells [137]. Cultures of various glioma cell lines (C6 and U138MG) revealed an increased expression and activity of this enzyme in relation to increased confluences of cells and the culture times. Adenosine is another factor engaged in progression of gliomas by activation of P1 receptors and thus induction of glioma cells proliferation [134], tumour growth, angiogenesis (A_2B_ receptor), inhibition of immune response (inhibition of Tc lymphocytes migration and neoplastic cells recognition) (A_2A_) [137], increase in U138MG glioma cells adhesion [143], as well as expression of IL-6 and IL-8 (A_2B_) in U87MG human glioma cells and expression of MMP-9 (metaloproteinase-9) (A_3_) in glioblastoma multiforme cells [137]. Apart from the above, ecto-5'-nucleotidase/CD73 plays a role of adhesion molecule and interacts with different components of the extracellular matrix, whereas laminin and chondroitin sulphate may have influence on the activity of this molecule and adhesion of glioma cells that depends on adenosine [143]. Utilising antagonists of P1 receptors, such as APCP (αβ-methylene ADP) prevents proliferation (also the AMP) [134] and adhesion of the U138MG cell line [143].

Summing up, purinergic signalling participates in the development and progression of gliomas mainly through ATP and adenosine, as their concentration in the extracellular space is high in relation to altered activity of ectonucleotidases and the fact that considerable amounts of nucleotides are being released from neighbouring, damaged cells. They activate specific receptors on surface of glioma cells, such as P2X_7_, various subtypes of P2Y receptors, especially P2Y_12_, and adenosine receptors. This results in increased production of chemokines, intensified proliferation of cells with attenuated differentiation capacity, increased tumour invasiveness and angiogenesis.

#### 2.5.2. Neuroblastoma (NB)

Neuroblastoma is a malignant neoplasia originating from the sympathetic nervous system, the second most common solid tumor in children. This malignant tumor derives from neuronal-crest-neuroectodermal cells and is characterized by heterogeneous pathology and diversity of clinical phenotypes [144,145,146]. The control of neuroblastoma progression involves a complex network of signaling pathways acting at specific locations and developmental stages, whose disruption leads to developmental aberrations and cancer [145]. These pathways involve the activation of tyrosine kinase receptors [147,148], which via a restricted set of signaling cascades can induce a variety of responses such as the transcription of genes controlling growth, migration, morphology, and survival of neurons [149,150]. About 50% of NB patients present with metastatic disease at diagnosis, and only one-third of them survive at five years despite surgery, radiotherapy and aggressive chemotherapy followed by autologous hematopoietic rescue [151]. Like most metastatic cancers, the probability of cure is low at <30%. At the initial diagnosis of Stage 4 neuroblastoma, dissemination to the bone marrow and extension to bone is nearly universal, whereas CNS metastasis is extremely rare [152,153].

In normal tissues that express P2 receptors, ATP is locally secreted in response to specific physiological signals but can also be released in a nonphysiological manner, such as at sites of mechanical stimulation, hypoxia, tissue injury or inflammation [154,155]. Extracellular ATP is generally considered the prototypical danger signal, as it accumulates at inflammatory sites and within the tumor interstitium at concentrations that may reach the hundred micromolar range. On the contrary, ATP is almost undetectable in the interstitium of healthy tissues [2,4]. Extracellular ATP is released into the tumor microenvironment by cancer cells and infiltrating inflammatory cells through different mechanisms including granule exocytosis, plasma membrane channels or lysis [156].

To date knowledge on the role of purinergic signaling in neuroblastoma progression and development is still very limited (Table 1). The biological role of P2 receptors in neuroblastoma is still neither very well characterized nor does a correlation exist between P2 receptor expression and prognosis in neuroblastoma. However, several studies were conducted showing important functions of nucleotides and purinergic receptor expression in neuroblastoma cell lines and animal models.

Early studies demonstrated P2X_7_ receptor expression on NG1O8-15 cells and their parent cell line, N18TG-2. These cell lines are mouse neuroblastoma (NG1O8-15) hybridized with rat C6BU-1 rat glioma cells. These cells expressed functional receptor with strong (92%) homology to mouse P2X_7_ receptor. The cells produced electrical current when the P2X_7_ receptor was stimulated by ATP and were sensitive to several purinergic receptor antagonists [71]. In further studies it was shown that the hybrid cell line and N1E 115, Neuro-2A, and NB4 1A3 mouse neuroblastoma cell lines robustly responded to UTP and UDP expanding the variety of purinergic receptors expression. However, only NG1O8-15 greatly responded to ATP. Thus, the pharmacological profile: UTP ≥ UDP ≫ UMP > ATP = ADP, was frequent in neuroblastoma cell lines [157]. Finally, RT-PCR study revealed P2Y_6_ and P2Y_4_ receptor expression [158] in previously mentioned cell lines. Probable expression of P2Y receptors on mouse neuroblastoma cell line (Neuro-2A) was first noted by Van Zoelen [159] and further evaluated by Chen *et al.* [160], when the cells incubated with UTP and UDP produced concentration-dependent increase in the accumulation of inositol phosphates. The effect was not halted by pertussis toxin (PTX), which demonstrated that the reaction was mediated by toxin-insensitive G_q_ protein. Supposedly, the P2Y_2_ receptor is PTX-insensitive and abundantly expressed on Neuro-2A cells; this was later demonstrated by Leon-Otegui [161]. Another murine neuroblastoma cell line that was extensively assessed in the context of P2Y receptor expression was NIE-115. Presence of the P2X_7_ receptor was demonstrated, and surprisingly, the receptor was not involved in triggering apoptosis. Thus, breakdown to adenosine was the main route for ATP-induced apoptosis [162].

P2Y expression was extensively evaluated in human neuroblastoma cell lines. In 2001 Moore *et al.* [163] screened more than 40 cell lines by Taq-man PCR. In the study high P2Y_4_ (SK-N-BE and SH-SY-5Y) and P2Y_11_ (NT-2, IMR32 and SH-SY-5Y) expression was noted; P2Y_1_, P2Y_2_, P2Y_6_ and P2Y_14_ were absent. In another study functional expression of the P2Y_4_ receptor on SH-SY-5Y cells was confirmed [33]. Similarly to murine neuroblastoma, P2X_7_ expression was very high in human cell lines and, what is very important, also in clinical specimens [72,73]. In the latter paper, it is very interesting how neuroblastoma cells benefit from P2X_7_ expression. First, it does not induce apoptosis by the inability to turn caspase-3 and second, it initiates release of growth promoting factors, like substance P. Recently, it was shown that neuroblastoma cell line NB NXS2, when injected into the mice released ATP at the sites of tumor dissemination, increasing in time [74]. Extracellular ATP released from neuroblastoma cells may act in paracrine/autocrine model driving tumor proliferation and, at the same time, activating myeloid-derived immunosuppressive cells that produce growth-promoting chemokines like CCL2 [74].

Stimulation of P2X_7_ receptor by ATP supports proliferation of neuroblastoma cells. What is more, as the neuroblastoma cell line SH-SY-5Y is characterized by high P2Y_4_ receptor expression, this receptor may be involved in neuronal differentiation of the cells, upon stimulation with UTP. Notably, the UTP/P2Y_4_ may act as an inducer of cell death after differentiation.

While nucleotide-induced apoptosis was previously considered to be mediated by ionotropic P2 receptors only [164,165], there is now growing evidence that by altering the intracellular calcium concentrations, metabotropic P2 receptors might also participate in both growth inhibition and programmed cell death [166,167,168,169]. The same cellular context was used when the P2Y_6_ receptor was overexpressed in SH-SY-5Y, and UDP exerted cytostatic actions and engaged apoptosis [87]. Similarly, neuritogenesis was described when murine Neuro2a cells were stimulated with ATP, through the P2Y_11_ receptor [170]. Neuro2A cells were also used for a very interesting experiment where P2X_7_ receptor antagonists were applied [75]; as a result, prominent neurite outgrowth was detected. The same result was achieved when down‑regulation by siRNA was applied. Retinoic acid-induced differentiated Neuro2a cells expressed P2X_7_ at significantly lower levels. The authors conclude that P2X_7_ receptors may play a critical role in maintaining Ca^2+^ homeostasis and cell survival of neuroblastoma cells [75].

There are several ways how extracellular nucleotides can influence cancer biology. We may envision that after gamma-irradiation or application of chemotherapeutics, which are standard methods in neuroblastoma treatment cancer cells, cells of immunological system or cells of bone marrow stroma start to release adenine nucleotides into tumor environment. Whether extracellular ATP accumulation will turn out to be beneficial or detrimental for the host will depend on the panel of P2 receptors expressed by the tumor cells and by the infiltrating inflammatory cells, the concentration, and the rate of degradation to adenosine.

## 3. Conclusions

Available results of studies focusing on the role played by purinergic signalling in nervous system disorders indicate that: (1) it participates in pathogenesis of nervous system diseases; (2) changes in the expression of P2X and P2Y receptors and ectonucleotidases, as well as increased concentrations of extracellular nucleotides may influence the course and progression of the disease; (3) characteristics of P2 receptors may be used in development of new therapies.

The P2X_7_ receptor is a particularly important subtype of a receptor. It is activated by high concentrations of ATP (>100 μM) occurring only in pathology [38]. In the nervous system P2X_7_ receptors are expressed in microglia, astrocytes, oligodendrocytes and neurons. The existence of P2X_7_ receptors on neurons was for a long time unconfirmed due to the poor specificity of antibodies and ligands used [171]. It is now clear that P2X_7_ receptors are expressed in neurons in different regions of brain [19,35,51,64,172]. The role of P2X_7_ receptors is emphasised in all diseases described in this review, mainly receptors derived from microglia and astrocytes. These cells play a crucial role in induction of neuroinflammation, which is an important factor in pathogenesis of neurodegenerative diseases [15,52,53,54,61,64], depression [47,49,50] or neuropathic pain [38,39]. The alterations in glial cell functions significantly affect neuron functions and may contribute to development of nervous system disorders. There are not many studies that indicate the role of neuronal P2X_7_ receptors. Probably the reason is the above-mentioned poor specificity of used reagents. This issue requires further research.

It is worth mentioning that purinergic signalling is a complex network of numerous receptors and nucleotides, as well as ectonucleotidases responsible for their degradation. Signalling mediated by adenosine, being a product of ATP degradation, is also of considerable importance. Hence, new therapeutic strategies should base their action not only on changing the activity of receptors (synthetic agonists/antagonists), but also on changing their expression and on manipulating the release and breakdown of extracellular nucleotides. Reports that have been presented in this review indicate only a small part of this wide issue and they certainly require further detailed investigation. Let us hope that future studies will lead to a more profound understanding of the mechanisms underlying nervous system diseases, and thus promote the development of effective neuroprotective agents.
